# Phenotypic variability in traits related to flight dispersal in the wing dimorphic species *Triatoma guasayana*

**DOI:** 10.1186/s13071-022-05570-7

**Published:** 2023-01-09

**Authors:** Gisel V. Gigena, Claudia S. Rodríguez, Federico G. Fiad, María Laura Hernández, Ana Laura Carbajal-de-la-Fuente, Romina V. Piccinali, Paz Sánchez Casaccia, Antonieta Rojas de Arias, Patricia Lobbia, Luciana Abrahan, Marinely Bustamante Gomez, Jorge Espinoza, Florencia Cano, Julieta Nattero

**Affiliations:** 1 Cátedras de Morfología Animal y de Introducción a la Biología, Instituto de Investigaciones Biológicas y Tecnológicas (IIByT), Facultad de Ciencias Exactas Físicas y Naturales, Consejo Nacional de Investigaciones Científicas y Técnicas (CONICET)/Universidad Nacional de Córdoba, Av. Vélez Sársfield 299, X5000JJC Córdoba, Argentina; 2 Consejo Nacional de Investigaciones Científicas y Técnicas (CONICET), Buenos Aires, Argentina; 3 Unidad Operativa de Vectores y Ambiente (UnOVE), Administración Nacional de Laboratorios e Institutos de Salud “Dr. Carlos Malbrán, Centro Nacional de Diagnóstico e Investigación en Endemo-Epidemias (CeNDIE), Santa María de Punilla, Córdoba, Argentina; 4 Centro Nacional de Diagnóstico e Investigación en Endemo-Epidemias (CeNDIE), Administración Nacional de Laboratorios e Institutos de Salud “Dr. Carlos Malbrán” (ANLIS), Av. Paseo Colón 568, Buenos Aires, Argentina; 5 Departamento de Ecología Genética y Evolución, Laboratorio de Eco-Epidemiología, Facultad de Ciencias Exactas y Naturales, Universidad de Buenos Aires, Intendente Güiraldes 2160, Ciudad Universitaria, Pabellón 2, C1428EGA Ciudad Autónoma de Buenos Aires, Argentina; 6 Instituto de Ecología, Genética y Evolución (IEGEBA), Intendente Güiraldes, CONICET/Universidad de Buenos Aires, 2160, Ciudad Universitaria, Pabellón 2, C1428EGA Ciudad Autónoma de Buenos Aires, Argentina; 7 Centro para el Desarrollo de la Investigación Científica (CEDIC), Manduvirá 635 entre 15 de agosto y Oleary, Asunción, Paraguay; 8 Centro Regional de Investigaciones Científicas y Transferencia Tecnológica de La Rioja (CRILAR), UNLAR, SEGEMAR, UNCa, CONICET, Entre Ríos y Mendoza S/N, Anillaco , 5301 La Rioja, Provincia de La Rioja Argentina; 9 Departamento de Apoyo y Asesoramiento a Proyectos, Universidad Privada del Valle, Campus Tiquipaya, Cochabamba, Bolivia; 10 Departamento de Biología, Laboratorio de Entomología Médica, Universidad Mayor de San Simón, Cochabamba, Bolivia; 11 Programa de Control de Vectores, Ministerio de Salud Pública de San Juan, San Juan, Argentina

**Keywords:** Chagas disease, Head, Pronotum, Wing, Linear morphometrics, Geometric morphometrics, Wing dimorphism

## Abstract

**Background:**

*Triatoma guasayana* is considered an emerging vector of Chagas disease in the Southern Cone of South America. The presence of a triatomine population with brachypterous individuals, in which both wings are reduced, has recently been reported for this species. The aim of the present study was to determine if flight-related traits varied across populations, if these traits could explain differences in flight capacity across populations and if flight-related traits are associated with geographic and/or climatic variation.

**Methods:**

The study involved 66 male *T. guasayana* specimens from 10 triatomine populations. Digital images of wing, head and pronotum were used to estimate linear and geometric morphometric variables. Variations in size and shape were analysed using one-way analysis of variance and canonical variate analysis (CVA), respectively. Mantel tests were applied to analyse the relationship between morphometric and geographic distances, and the association between size measurements was analysed using Pearson’s correlation. We explored covariation between size and shape variables using partial least square analyses (PLS). The association of geographic and climatic variables with size measurements was tested using linear regression analyses. We performed PLS analyses for shape measurements.

**Results:**

Wing size differed significantly across triatomine populations. The CVA showed that wing shape of the brachypterous population is well discriminated from that of the other populations. The Mantel test showed a positive and significant association between wing shape and geographic distances. The heads of the brachypterous population were significantly larger than those of the other populations. Similar to wing shape, the head shape of the brachypterous population was well discriminated from those of the other populations. Pronotum width did not show significant differences across populations. Geographic and climatic factors were associated with size and shape of both the wing and head, but not with pronotum width.

**Conclusions:**

Most of the traits related to flight dispersal varied across populations. Wing shape and head shape were found to be better markers for differentiated morphological variation across populations. Head measurements also varied in accordance with this condition. Geographic and climatic variables were associated with most of the flight-related traits.

**Graphical Abstract:**

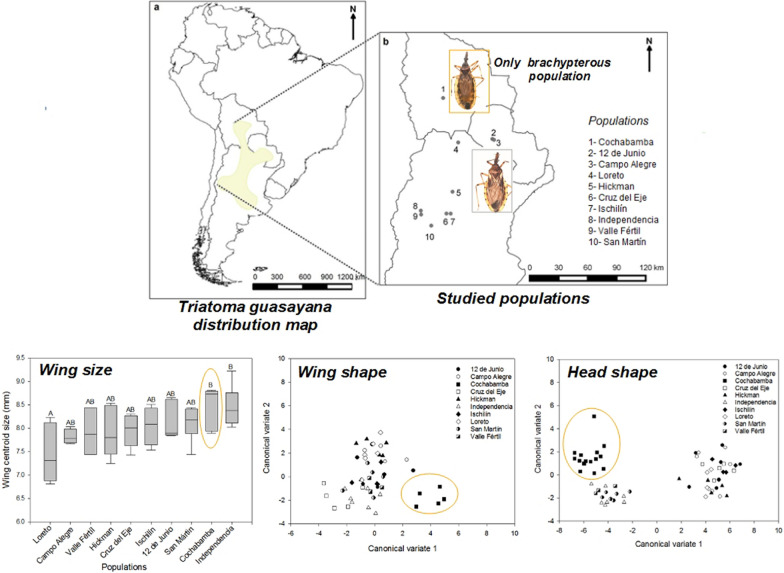

**Supplementary Information:**

The online version contains supplementary material available at 10.1186/s13071-022-05570-7.

## Background

In many organisms, environmental heterogeneity and ecological gradients can generate phenotypic variation [1]. Phenotypic patterns of morphological variations within insect species might reflect different dispersal patterns and habitat availability. Intraspecific morphometric analyses should focus on samples from a broad geographical range to understand variations in morphometry of individuals that occur in diverse environments. Additionally, to understand how environmental heterogeneity contributes to changes in phenotypic variation, knowledge on whether some morpho-functional mechanisms generate this pattern of variation is desirable [2].

The subfamily Triatominae (Hemiptera: Reduviidae) includes hematophagous insects that act as vectors of Chagas disease, one of the most important parasitic diseases in Latin America [3]. Currently, there are 157 living species [4] of triatomines, which differ in their epidemiological importance and in aspects of their biology [5]. In recent years, several studies have reported the occurrence of sylvatic triatomines dispersing actively to domestic environments in Argentina, Brazil and Paraguay [6–8]. Although sylvatic species are considered to be of secondary epidemiological importance, they are responsible for maintaining the sylvatic transmission cycle of *Trypanosoma cruzi* [9].

*Triatoma guasayana* is a sylvatic species that invades peridomestic and domestic habitats. It is distributed in the dry western Chaco region of Argentina, Bolivia and Paraguay. Some laboratory and field studies have shown that this triatomine species has vectorial capacity [8, 10–13]. In sylvatic ecotopes, *T. guasayana* has been found in dry cacti, bromeliads and fallen dry logs, and in peridomestic habitats, it is associated with chicken coops, goat or sheep corrals, piled materials and orchard fences [14–16]. *Triatoma guasayana* is traditionally considered to be part of the “sordida subcomplex” [17]. However, phylogenetic analysis indicated that *T. guasayana* is more closely related to the “rubrovaria subcomplex” than to members of the “sordida subcomplex” [18, 19]. According to Gorla and Noireau [20], *T. guasayana*, among other species, may be considered an emerging vector in the Southern Cone of South America.

It has been postulated that flight dispersal is the main mechanism of invasion, infestation and/or recolonization of houses by adult triatomines [21, 22]. Nutritional and reproductive status as well as population density and environmental conditions are known factors that modulate flight dispersal in member of the Triatominae [23–26]. There are interspecific differences in terms of dispersal capacity by flight; for example, the number of flying individuals was found to be higher in *T. guasayana* than in *T. infestans* and other secondary triatomines [12]. Flight dispersal may vary markedly even within the same species. Some species present polymorphisms in wing length and/or in the development of the flight muscles that affect the active dispersal capacity [27, 28]. Wing polymorphism implies variation in the size of the wings or in their presence/absence [27]. The diversity of wing morphotypes is usually associated with other morphological attributes, such as head shape, thorax size and olfactory capacity. In *Mepraia spinolai*, a species with wing dimorphism, macropterous individuals (with normal wing size) exhibited a greater development of the thorax than micropterous specimens [29]. For *T. guasayana*, the presence of a population with brachypterous individuals, in which both wings are reduced, has recently been reported [30]. In this study, changes in the shape of the head according to wing characteristics were also determined, with macropterous individuals found to have a shorter head and a greater distance between eyes [30]. These observations in *M. spinolai* and *T. guasayana* suggest that the varieties of wing morphotypes imply a diversity of body structures. Polymorphism in flight muscles has been reported and includes developmental changes that affect dispersal capacity [31]. In some triatomine species, a relationship between wing size and thorax development has been demonstrated. Hybrids between *Triatoma sherlocki* and *Triatoma juazeirensis* have an increased dispersal capacity that coincides with a wider thorax, despite having wings of intermediate size. A wider thorax may imply greater development of the wing muscles. *Mepraia spinolai* showed an increase in thorax size as a function of wing development [29].

Taking into account the existence of wing dimorphism in *T. guasayana* and that this condition determines flight functionality, the aim of this study was to understand: (i) if traits related to dispersal characteristics varied across populations; (ii) if size and/or shape variation of flight-related traits could explain differences in flight capacity across populations; and (iii) if flight-related traits are associated with geographic and/or climatic variation.

## Methods

### Insects

**Fig. 1:**
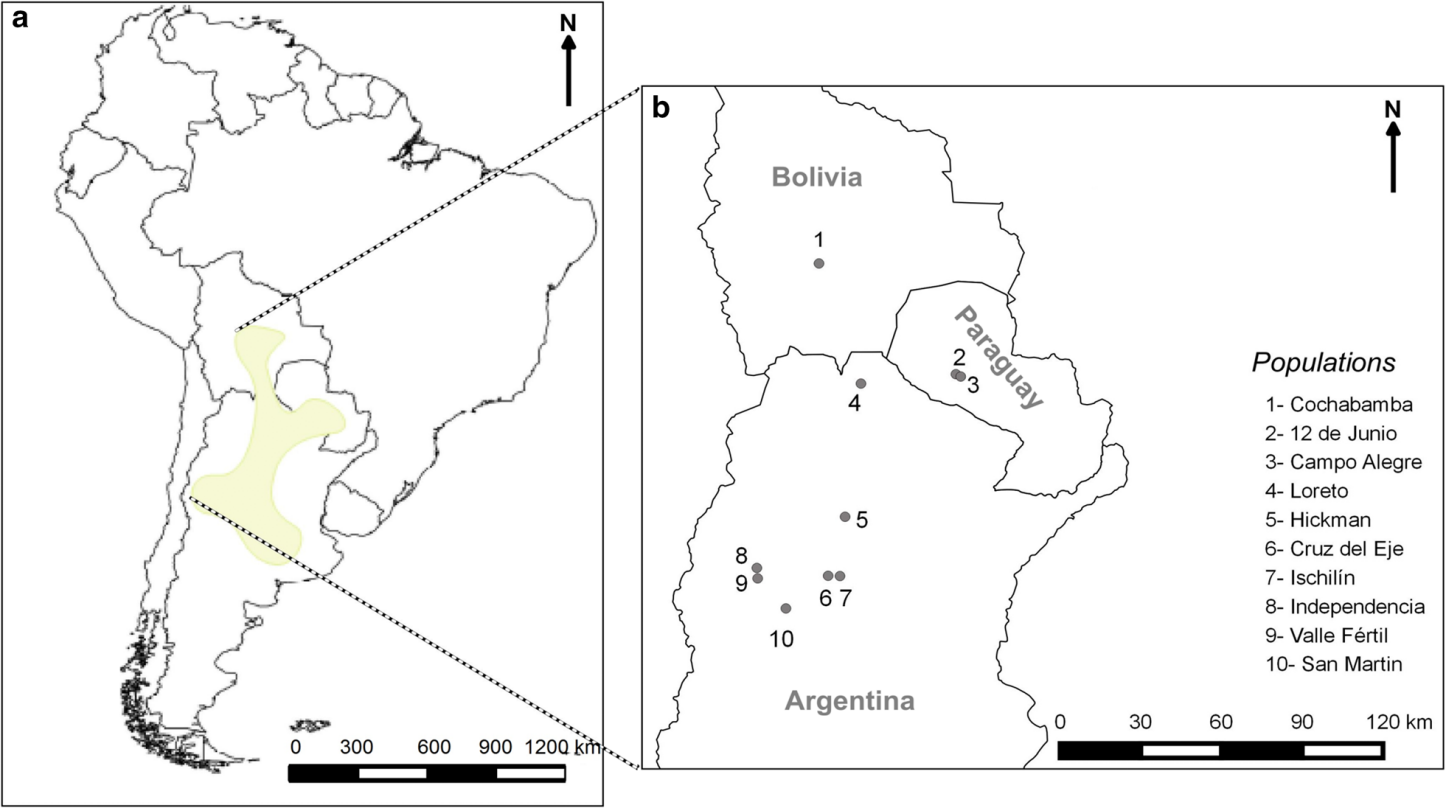
**a **Distribution area of *Triatoma guasayana* based on [63]. **b** Location of the study populations

**Fig. 2:**
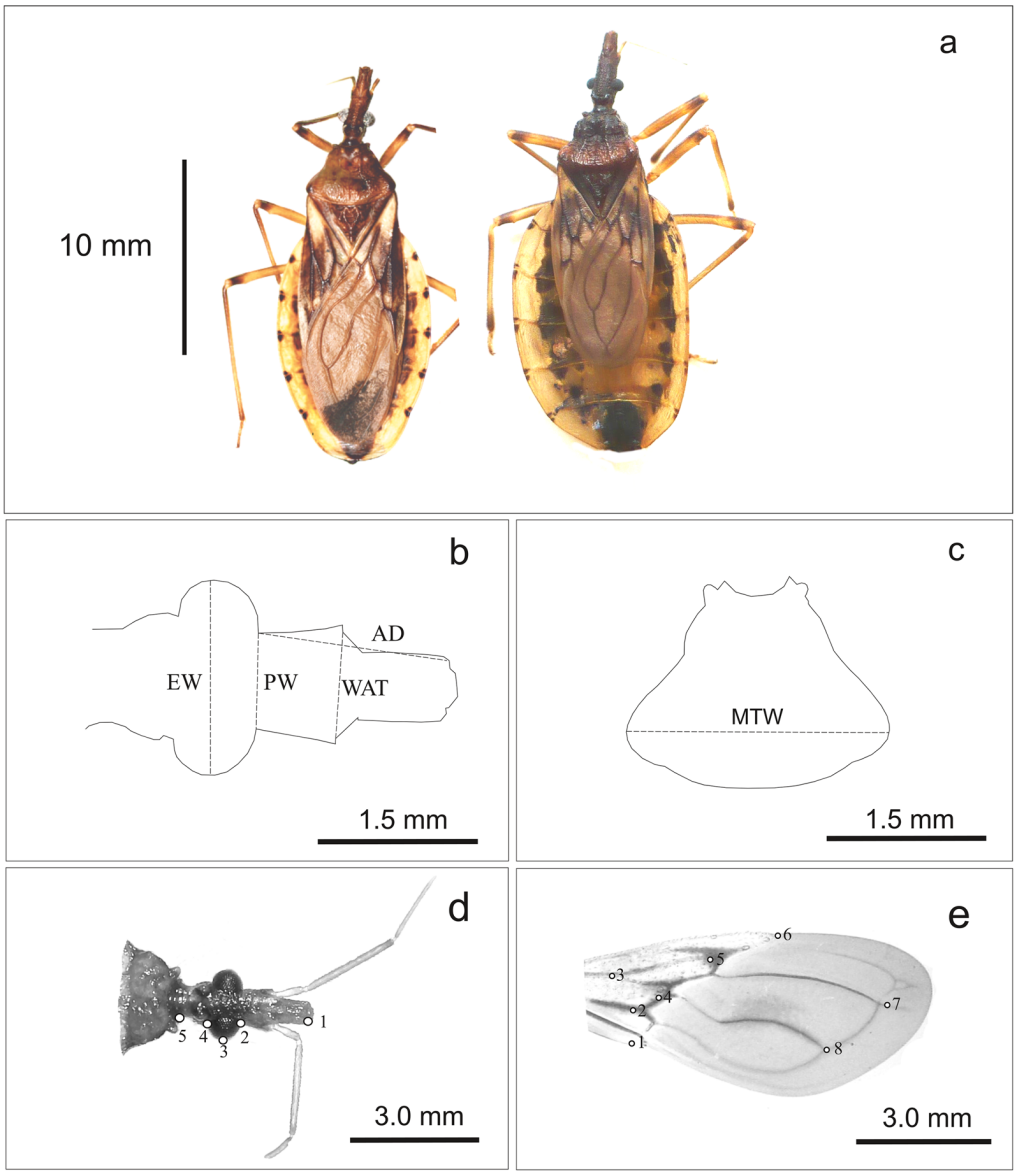
**a** Specimens of macropterous (left) and brachypterous (right) *Triatoma guasayana*. **b**–**e** Linear measurements of the head (**b**) and pronotum (**c**) and landmark position for the head (**d**) and wing (**e**) in the *T. guasayana* populations included in this study. Head: AD, Anteocular distance; EW, maximum width across the eyes; MTW, maximum thorax width; PW, preocular width; WAT, width between antenniferous tubercle (WAT)

The study involved 66 males from 10 populations of *T. guasayana* (Table 1; Fig. 1). Only males were studied because the sample included more males than females, and because other studies suggest sexual dimorphism ([30] and J Nattero, unpublished data). For the Cochabamba population, from the 16 collected males, wings of five individuals could be included since the remaining individuals had not been properly preserved. All populations included in this study were collected from peridomiciliary structures. All adults from the Cochabamba population are brachypterous, a condition first reported by Hernández et al. [30] (Fig. 2a). For this population, the dispersal capacity in the field was evaluated with light traps, active searching and live-bait mouse traps (also known as Noireau traps). Although individuals were captured by active searching and with Norieau traps, no individuals were captured with light traps (J Espinoza, personal communication). A number of assays were carried out in the laboratory to verify the mode of dispersal; only dispersal by walking was observed (J Espinoza, personal communication). Atrophy of the alar muscles in the thoracic box was also observed in a few inspected individuals (ML Hernández, personal communication). We took digital images of the dorsal view of head, pronotum and right wing using digital cameras (Lumix DMC-ZS7, Panasonic Corporation, Kadoma, Osaka, Japan; and Nikon S9900, Nikon Corporation, Tokyo, Japan) attached to a stereomicroscope (Stemi SV-11; Carl Zeiss, Wetzlar, Germany) at 6× magnification. All images included a reference scale.

**Table 1:** Geographical location, coordinates and individual number of collected *Triatoma guasayana* individuals at each collection site

Population	Province/state	Country	Geographical variables			Number of individuals
			Latitude (S)	Longitude (W)	Altitude (m a.s.l.)	
Cruz del Eje	Córdoba	Argentina	− 30.57	− 64.81	431	5
Independencia	La Rioja	Argentina	− 30.27	− 67.46	1029	8
Ischilín	Córdoba	Argentina	− 30.58	− 64.37	900	4
Hickman	Salta	Argentina	− 23.21	− 63.59	261	5
Loreto	Santiago del Estero	Argentina	− 28.31	− 64.18	140	6
Valle Fértil	San Juan	Argentina	− 30.67	− 67.43	879	3
San Martin	La Rioja	Argentina	− 31.82	− 66.38	508	6
Cochabamba	Cochabamba	Bolivia	− 18.61	− 65.15	1380	16
12 de Junio	Presidente Hayes	Paraguay	− 22.94	− 59.88	128	7
Campo Alegre	Boquerón	Paraguay	− 22.85	− 60.06	138	6
Total						66

### Geographic and climatic characterization

To obtain the geographic location for each population, we recorded latitude, longitude and altitude. The geographical coordinates of the 10 populations were imported into the Google Earth Engine platform to extract the climatic variables for each site. We used ERA5 (Copernicus Climate Data Store; https://cds.climate.copernicus.eu/#!/home) to obtain the climate data with a spatial resolution of 11 × 11 km. Of the 50 climate variables available, in this work we only included data from annual rainfall and annual mean temperature. We also considered the average monthly dew point temperature and the average monthly temperature and estimated the relative humidity (RH), including these data as a third variable to characterize climate variation. RH was calculated as [32]:

$$RH =100 -5 x (T -Td)$$

where RH indicates relative humidity, *T* represents the average monthly temperature and *Td* is the average monthly dew point temperature. We only included these three environmental variables because they are determinants of flight dispersal in insects [33, 34].

### Linear morphometry

Linear measurements of the head and pronotum were made using the free software package tpsDig2 version 2.31 (http://life.bio.sunysb.edu/morph/). The linear measurements of the heads included anteocular distance (AD), width between antenniferous tubercles (WAT), preocular width (PW) and maximum width across the eyes (EW) (Fig. 2b). For the pronotum, we measured the maximum thorax width (MTW) (Fig. 2c). All linear measurements showed a normal distribution.

### Geometric morphometry

A landmark-based approach was applied to study geometric morphometrics of the heads and right forewings. We defined and collected five coplanar type II landmarks of the ventral view of the head, and eight type I landmarks of the wings using the software package tpsdig2 (Fig. 2d, e). We extracted data on head and wing shape with a generalized full Procrustes fit and a projection to shape tangent space [35]. We used Procrustes coordinates as shape variables. Centroid size (CS; i.e. the square root of the sum of squared distances from each landmark to the centroid of the configuration) was computed as a measure of wing size and head. All of these steps of the morphometric analysis were performed using the free software MorphoJ version 1.07a [36].

### Statistical analysis

Linear and geometric variables of the head, pronotum and wings were analysed separately. Variations across populations for the single linear measurements of the wing, head and pronotum were explored via one-way analysis of variance (ANOVA) and Tukey’s post-hoc tests for each variable, using the software InfoStat version 2016 [37].

For shape measurements of the head and wings, we calculated Procrustes distances between pairs of populations and evaluated their significance via a non-parametric test based on permutations (1000 runs), using MorphoJ [36]. We then performed canonical variate analyses (CVA) and calculated the percentage of phenotypic similarity between pairs of populations using the cross-check test of discriminant analysis [38] in InfoStat software [37]. The Procrustes distances were represented in unrooted neighbour-joining (NJ) trees using the free software MEGA X 10.2.6 [39]. We calculated bootstrap values for 1000 replications following Ascarranuz et al. [40]. The relationship of geographic distances with linear and geometric morphometric measurements of head, pronotum and wing was analysed using Mantel tests with the free software PASSAGE 2 2.0.11.6 [41]. To construct the morphological distance matrices between populations, we used Procrustes distances for shape measurements and Euclidean distances for size measurements.

The association between wing, head and pronotum size was explored via a Pearson’s correlation, using InfoStat software [37]. We analysed the covariation between wing, head and pronotum shape and between size and shape using partial least square analyses (PLS) in MorphoJ [36].

To summarize the variation and describe the environmental characterization of the six geographic and climatic factors (i.e. latitude, longitude, altitude, annual rainfall, annual mean temperature and RH), we used a principal component analysis (PCA) with R package FactorMineR 2.4 [43]. The first two principal component (PC) scores described 95.04% of the total variance (59.72–35.69% for PC1 and PC2, respectively). In PC1, the factors with relevant coefficients (> 0.7) were latitude, altitude, temperature, precipitation and RH (Additional file 1: Table S1). In PC2, only longitude was an important factor (Additional file 1: Table S1). We explored the association between PC1 and PC2 and measurements of the head, wing and pronotum size across the 10 populations studied using a multiple linear regression analysis [43]. The covariation between geographic and climate variables and wing and head shape variation was explored using PLS.

## Results

### Changes in wing size across populations

**Fig. 3:**
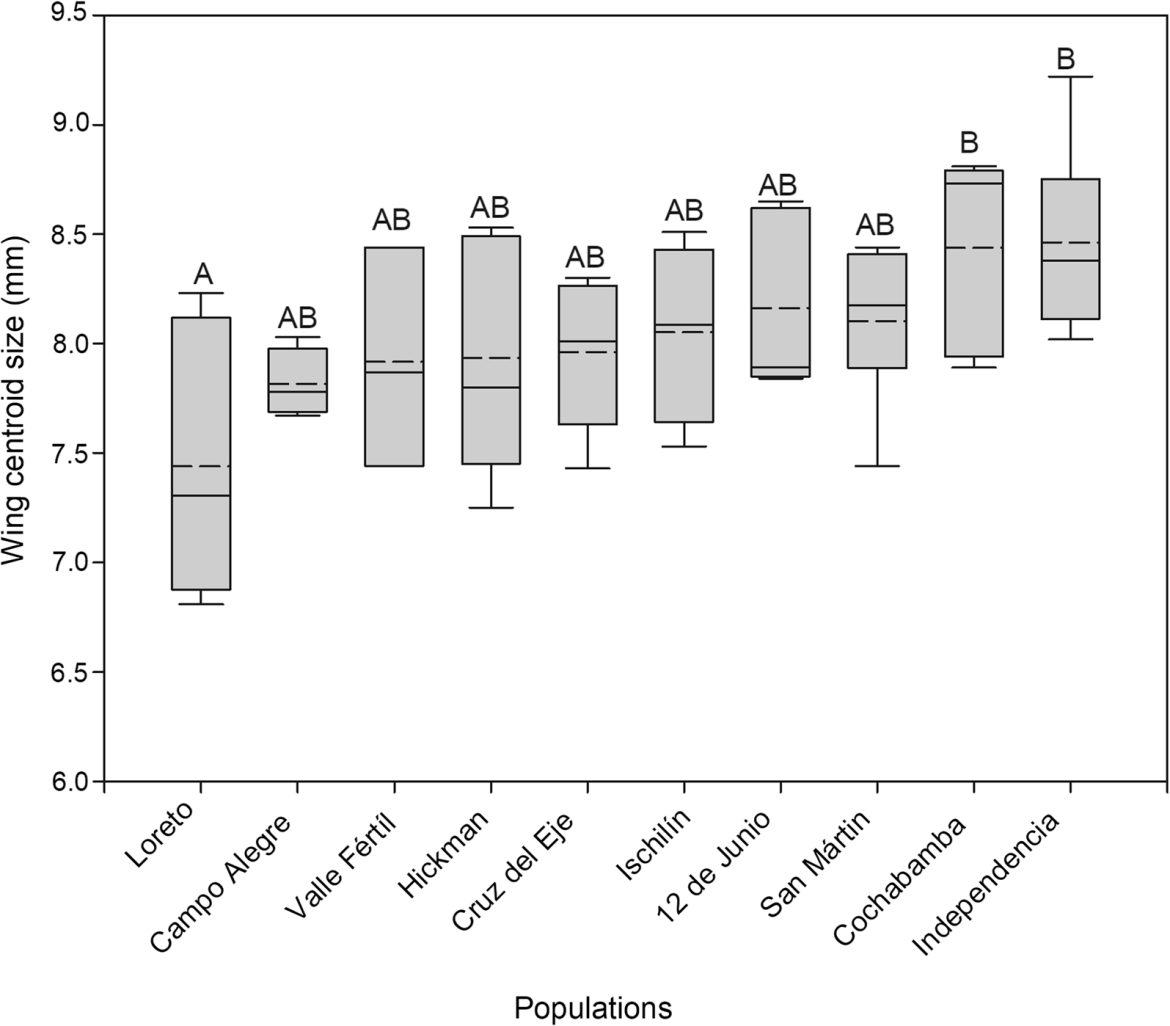
Box plot (median and standard deviation) of wing centroid size across populations of *T. guasayana*. Solid horizontal line represents the median value; dashed horizontal line, the median value; the whiskers, the standard deviation. Different uppercase letters above boxes indicate statistical difference at *P* < 0.05 (Tukey’s post-hoc tests)

Significant differences in wing size were observed among populations (*F*_(9, 42)_ = 2.99, *P* = 0.008) (Fig. 3). Tukey’s post-hoc tests showed significant differences between the Loreto population and two other populations: Cochabamba, the brachypterous population, and Independencia (*P* < 0.05) (Fig. 3). The Loreto population presented a smaller wing size than the Cochabamba and Independencia populations, whereas the latter two had the largest wings of the 10 populations. The Mantel test showed a negative and significant association between wing size and geographic distances (*Z* = 73,661.06, *r* = − 0.05, *P* = 0.04), indicating that the larger the differences in wing size, the smaller the geographic distance.

### Wing shape of the brachypterous population differs from that of the other populations

**Fig. 4:**
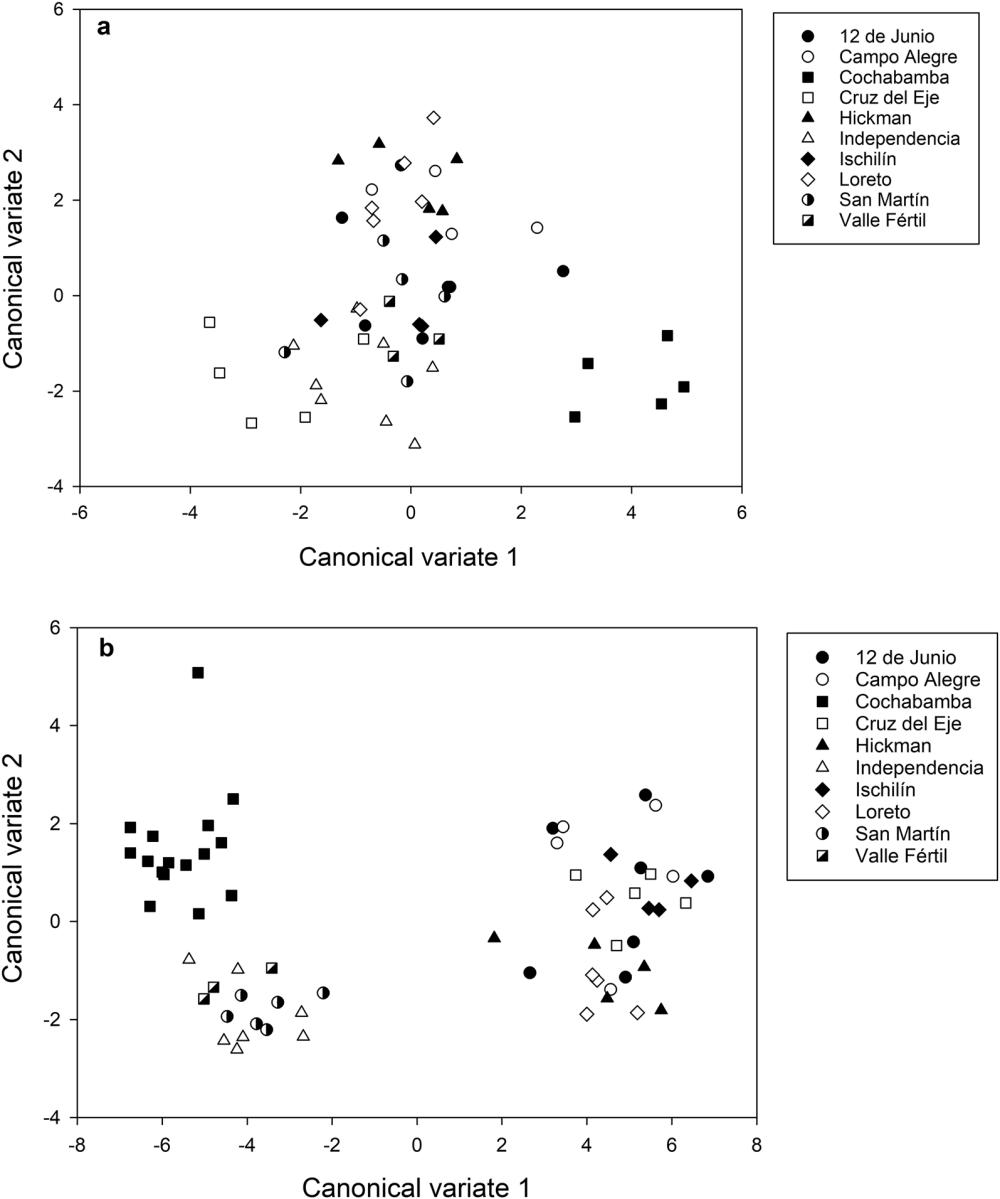
Factorial maps in the plane of the two first axes of the canonical variate analysis for shape measurements of wings and heads for populations of *T. guasayana*.** a** Wings,** b** heads

**Fig. 5:**
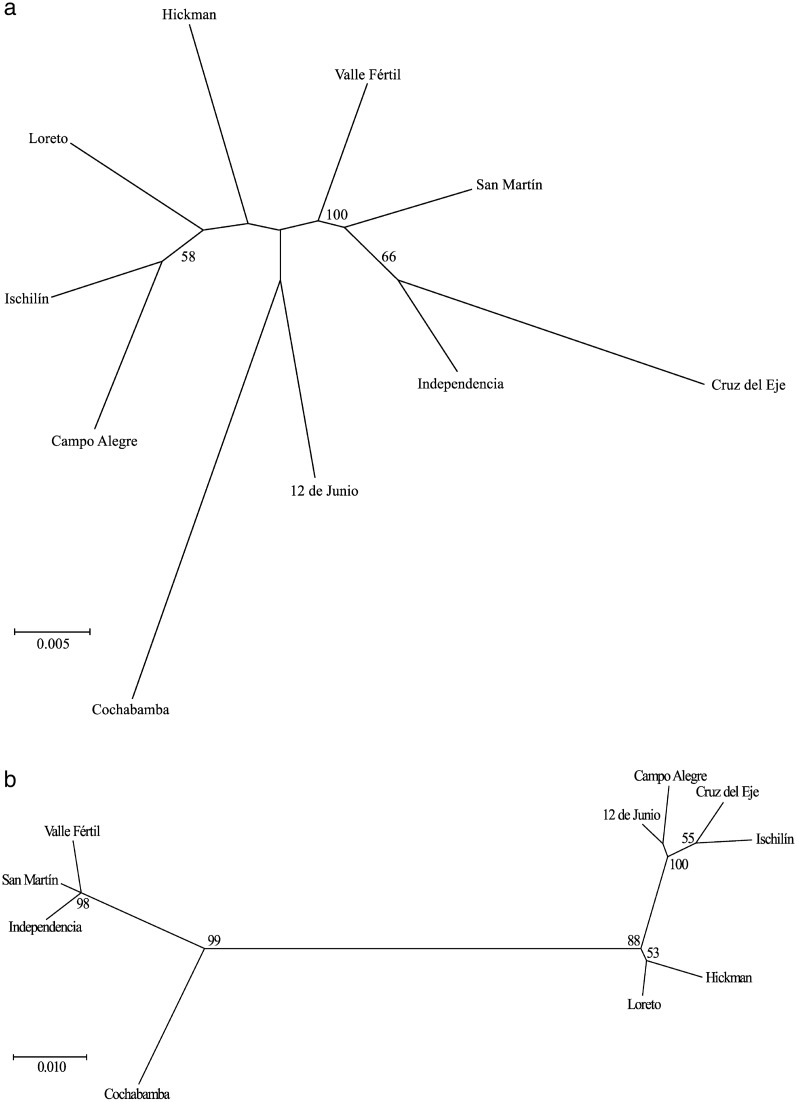
Neighbour-joining trees based on Procrustes distances of shape measurements for wings (**a**) and heads (**b**) for populations of *T. guasayana*. Numbers close to the nodes are 1000 replicates of bootstrap values

Wing shape differed among populations. The first two discriminant factors of CVA (CV1, CV2) explained 68.13% of the total variation (CV1: 42.60, CV2: 25.53%). The brachypterous population, Cochabamba, was found to be well discriminated from the other populations in the space of the first discriminant factor (CV1) (Fig. 4a). All individuals from this population, as well as those of the Campo Alegre, Cruz del Eje, Ischilín and Valle Fértil populations, were correctly assigned (0% of misclassified individuals) (Additional file 1: Table S2). In contrast, the San Martín population exhibited 60% of misclassified individuals (Additional file 1: Table S2). Procrustes distances between populations were significantly different (*P* < 0.05) for all of the comparisons that included the Cruz del Eje and Cochabamba populations (Additional file 1: Table S3). The NJ tree based on Procrustes distances is presented in Fig. 5a, which shows that the only bootstrap-supported clade was the one that included the Independencia and Cruz del Eje populations and the Ischilín and Campo Alegre populations (Fig. 5a). The Mantel test showed a positive and significant association between wing shape and geographic distances (*Z* = 2483.882, *r* = 0.44, *P* = 0.001), indicating that the smaller the differences in wing shape, the smaller the geographic distances.

### Head size of the brachypterous population is larger than that of the other populations

The ANOVAs performed for the four head linear measurements showed significant differences in all cases (Table 2). Tukey’s post-hoc tests showed that the brachypterous population had the highest values for all four measurements, although these were significantly different from those of the other nine populations only for the anteocular distance (*P* < 0.05) (Table 2). In the Mantel test, head size showed a positive and significant correlation with geographic distances (*Z* = 83,069.24, *r* = 0.324, *P* = 0.001,) indicating that the smaller the differences in head size, the smaller the geographic distance.

**Table 2:** Mean values and analysis of variance results between populations for linear head measurements of *T. guasayana*

Results	Anteocular distance	Width between antenniferous tubercles	Preocular width	Maximum width across the eyes
ANOVA between populations	*F*_(9.53)_ = 20.71, *P* < 0.0001	*F*_(9.53)_ = 5.01, *P* < 0.0001	*F*_(9.53)_ = 8.60, *P* < 0.001	*F*_(9.53)_ = 5.06, *P* < 0.0001
*Populations*				
12 de Junio	1.82 ± 0.06^a^	0.71 ± 0.03^a,b^	0.88 ± 0.03^a^	1.75 ± 0.05^a^
Campo Alegre	1.90 ± 0.07^a^	0.72 ± 0.03^a,b^	0.90 ± 0.03^a^	1.81 ± 0.05^a,b^
Cochabamba	2.48 ± 0.04^b^	0.83 ± 0.02^b^	1.06 ± 0.02^b^	2.02 ± 0.03^b^
Cruz del Eje	1.83 ± 0.07^a^	0.68 ± 0.03^a^	0.89 ± 0.03^a^	1.80 ± 0.05^a,b^
Hickman	1.82 ± 0.07^a^	0.75 ± 0.03^a,b^	0.94 ± 0.03^a,b^	1.86 ± 0.05^a,b^
Independencia	2.04 ± 0.05^a^	0.68 ± 0.02^a^	0.96 ± 0.02^a^	1.90 ± 0.04^a,b^
Ischilín	1.87 ± 0.08^a^	0.68 ± 0.03^a^	0.89 ± 0.03^a^	1.78 ± 0.06^a,b^
Loreto	1.78 ± 0.07^a^	0.70 ± 0.03^a,b^	0.93 ± 0.03^a^	1.77 ± 0.05^a^
San Martin	2.08 ± 0.06^a^	0.71 ± 0.03^a,b^	0.96 ± 0.03^a,b^	1.97 ± 0.05^a,b^
Valle Fértil	1.87 ± 0.09^a^	0.68 ± 0.04^a^	0.90 ± 0.04^a,b^	1.80 ± 0.07^a,b^

Values in table, other than the ANOVA values, are presented as the mean ± standard error (SE). Different lowercase letters indicate values within columns are statistically different at *P* < 0.05 (Tukey’s post-hoc tests)

The number of individuals analysed for all the measured traits are reported in Table 1

*ANOVA* Analysis of variance

### Head shape of the brachypterous population is different from that of the other populations

The first two discriminant factors accumulated 96.72% of the total variation (90.18 and 6.62% for the first and second axes, respectively). The space delimited by the first two discriminant factors showed three well-delimited groups, of which one included only the Cochabamba population, the brachypterous population, one included the Independencia, San Martín and Valle Fértil populations and the third included the remaining populations (Fig. 4b). All individuals of the Cochabamba and Ischilín populations were correctly assigned (0% of misclassified individuals) (Additional file 1: Table S2), while The population from 12 de Junio exhibited the highest number of misclassified individuals (85.71%) (Additional file 1: Table S2). Procrustes’ distances between populations were significantly different (*P* < 0.05) for all pairs of populations that included Cochabamba (Additional file 1: Table S3). The NJ tree based on Procrustes distances is presented in Fig. 5b. Bootstrap values supported different groups from different geographic areas; in particular, the brachypterous population was in a group that was separated from the rest (Fig. 5b). The Mantel test showed a positive and significant association between head shape and geographic distances (*Z* = 4499.254, *r* = 0.19, *P* = 0.005), indicating that the smaller the differences in head shape, the smaller the geographic distance.

### Pronotum width is similar across populations

The ANOVA for the pronotum width did not show significant differences across populations (*F*_(9, 51)_ = 1.78, *P* = 0.094). The Mantel test showed a negative and significant correlation with geographic distances (*Z* = 71,936.392, *r* = − 0.147, *P* = 0.024), indicating that the larger the differences in pronotum width, the smaller the geographic distance.

### Wing, head and pronotum measurements are associated

Pearson’s correlations showed positive and significant associations between wing, head and pronotum size measurements (Table 3). The PLS analyses showed no covariation between wing and head shape, wing shape and head size, or between wing shape and pronotum width (Table 3). Head shape showed significant covariation with pronotum width and wing size (Table 3).

**Table 3:** Results of Pearson’s correlation between linear measurements of wing, head and pronotum, and partial least square analyses between shape measurements of wing and head and between size and shape measurements of wings, head and pronotum in *T. guasayana* populations

Morphometric traits	Head shape	Head centroid size	Pronotum width
Wing shape	*r* = 0.51, *P* = 0.730	*r* = 0.28, *P* = 0.812	*r* = 0.41, *P* = 0.262
Wing centroid size	*r* = 0.46, *P* = 0.018	*r*_(51)_ = 0.57, *P* < 0.0001	*r*_(48)_ = 0.53, *P* = 0.0001
Pronotum width	*r* = 0.39, *P* = 0.047	*r*_(60)_ = 0.45, *P* = 0.0003	–

### Linear and shape measurements are associated with geographic and climatic variables

Multiple linear regression analysis showed that head linear measurements were significantly associated with geographic and environmental factors with both PC1 and PC2 coefficient (with the exception of width between antenniferous tubercles with PC2) (Table 4). In contrast, pronotum width and wing size were not associated with geographic or climatic factors (Table 4). PLS analysis showed significant covariation of head and wing shape with all geographic and climatic variables (Table 5).

**Table 4:** Results of multiple linear regression analysis of morphometric traits and geographic and climatic variables in *T. guasayana* populations

Morphometric traits	Coefficient						*r*^2^	*P*-value
	PC1 ± SE	*P*-value	PC2 ± SE	*P*-value	Intercept ± SE	*P*-value		
Anterocular distance	0.112 ± 0.012	< 0.000	− 0.047 ± 0.016	0.005	2.045 ± 0.023	< 0.000	0.606	< 0.000
Preocular width	0.027 ± 0.004	< 0.000	− 0.013 ± 0.005	0.024	0.951 ± 0.008	< 0.000	0.425	< 0.000
Width between antenniferous tubercles	0.025 ± 0.005	< 0.000	0.008 ± 0.006	0.206	0.732 ± 0.009	< 0.000	0.327	< 0.000
Maximum width across the eyes	0.029 ± 0.008	< 0.000	− 0.026 ± 0.010	0.013	1.874 ± 0.015	< 0.000	0.245	< 0.000
Pronotum width	0.048 ± 0.027	0.079	− 0.030 ± 0.0247	0.230	4.244 ± 0.042	< 0.000	0.068	0.102
Wing size (centroid size)	0.075 ± 0.051	0.151	− 0.074 ± 0.042	0.088	8.137 ± 0.076	< 0.000	0.067	0.087

*PC* Principal component

**Table 5:** Results of the partial least square analyses of wing shape and head shape with geographic and climatic variables in *T. guasayana* populations

Morphometric traits	Geographic variables			Climatic variables		
	Latitude	Longitude	Altitude	Temperature	Rainfall	Relative humidity
Wing shape	*r* = 0.72 *P* < 0.001	*r* = 0.55 *P* = 0.005	*r* = 0.63 *P* = 0.0003	*r* = 0.63 *P* < 0.001	*r* = 0.75 *P* < 0.001	*r* = 0.62 *P* < 0.001
Head shape	*r* = 0.56 *P* < 0.001	*r* = 0.74 *P* < 0.001	*r* = 0.78 *P* < 0.001	*r* = 0.72 *P* < 0.001	*r* = 0.56 *P* < 0.001	*r* = 0.54 *P* = 0.001

## Discussion

*Triatoma guasayana* exhibits a good dispersal capacity. In a study comparing this species with other triatominae species (*T. infestans*, *T. platensis* and *T. eratyrusiformis*) from the arid Chaco of Argentina, *T. guasayana* was found to have the highest average number of flying individuals [12]. Adult *T. guasayana* bugs may invade a wide variety of ecotopes and have a high potential for night flight dispersal during the hot dry months (August-November) [21, 44]. Since dispersal is linked to flight, the existence of wing dimorphism at the population level provides a suitable model to study not only evolutionary patterns, such as dispersal processes and population structure [45, 46], but also a range of ecological factors, including habitat structure and climate [47, 48].

Our results showed differences in wing size across *T. guasayana* populations. Although all triatomine males collected from Cochabamba showed a brachypterous condition, individuals from this population as well as those of the Independencia population exhibited a bigger wing size than the macropterous populations. Individuals from the brachypterous population had significantly greater body size than individuals from the two macropterous populations of Ischilín and Cruz del Eje (*F*_(2,11)_ = 6.76, P = 0.012). This result suggests that the individuals from the Cochabamba population are larger and that their body is comparatively larger than their wings, showing the presence of brachyptery in this population. The same tendency has been observed in other insects, such as in carabids, with brachypterous individuals exhibiting a larger body size than their macropterous counterparts. This variation is attributed to differences in dispersal strategies, taking into account that brachypterous individuals disperse by running [49].

Regarding wing shape, our results showed differentiation between the brachypterous population and the other nine populations included in this study. This result suggests that differences in wing shape are associated with the brachyptery condition, and that this pattern could be influenced by the differences in flight capacity across populations. Differences in the shape but not in the size of a structure are considered to be determined by genetic processes [50]. Our results also showed that there is no covariation between wing shape and size measurements of the wing, head and pronotum. These results suggest a lack of functional integration (i.e. coordination among parts) of wing shape and size measurements across populations, and may be related to wing dimorphism. Studies combining biomechanics of flight and morphometric approaches could contribute to a better understanding of the functional implications of morphological changes associated with wing dimorphism [61].

Insects use a combination of sensory inputs, including cephalic sensilla, ocelli, antennal sensilla and eyes, to control flight activity [52–54]. Our results show that, in general terms, the brachypterous population presents a larger head size than the remaining populations. For *T. guasayana,* anterocular distance and distance between eyes have been suggested as the differences in the head that could be associated with flight capacity; it has also been proposed that shorter heads are related to changes in the position of the ocelli [30]. We found significantly longer head measurements for the brachypterous population, which agrees with the association suggested by [30]. Regarding head shape, our results showed that the head shape of the brachypterous population differed from the that of the other nine populations. The NJ tree for Procrustes distances showed similarities between populations that are geographically close.

Flight muscles are located in the pronotum. Our results showed that pronotum width was the only measured trait that did not differ between populations. There are alternative, non-mutually exclusive explanations that might account for this result. First, among insects, there is muscle polymorphism associated with the degree of muscle development. A greater pronotum width does not necessarily indicate a greater development of these muscles, as has been reported for other insect species [55]. Alary polymorphism is not necessarily accompanied with flight muscle development [30]. Second, there are functional or developmental constraints that might limit the potential for evolutionary change of pronotum width and reduce the potential of the population to respond to selection or to undergo non-adaptive evolution by drift [56].

The association of morphological distance with geographic distances between populations showed contrasting results. While wing shape and head size and shape exhibited positive associations, wing and pronotum size showed negative associations. Thus, these results might imply geographic separation as a key factor in morphological differentiation of genetic exchange between populations. A positive association would imply that geographically closer populations are more similar to each other. It would also indicate morphological spatial structuring for wings and heads between the populations of this species. This positive spatial association would adjust to a model of isolation by distance, in which differentiation increases with increasing distance. By contrast, the sizes of the wing and pronotum showed negative associations, implying that the greater the distance between populations, the smaller the difference in wing and pronotum size, respectively. These results should be interpreted with caution. In some cases, the correlation values were low, such as those for wing size and shape and for pronotum width.

The association of geographic and climatic variables with size and shape measurements showed that the former variables better explain head size, head shape and wing shape than the other measurements. For wing size and pronotum width, only longitude showed association. This means that, considering geographic variables, all morphological measures followed an east–west variation, and that head size, head shape and wing shape additionally followed a north–south variation and an altitudinal variation. Considering the broad-scale geographic variation of the sampled populations, variation in geographic variables also determines variation in climatic conditions. Wing and head shape exhibit significant phenotypic plasticity in response to developmental temperature, and this plastic response (hot-to-cold) mirrors a pattern of geographic differentiation, as might be expected under a synergistic scenario of adaptive plasticity and adaptive genetic divergence [57, 58]. Similar results were found in the yellow dung fly *Scathophaga stercoraria* (Diptera: Scathophagidae) from the New World, with clines of variation associated with wing shape but not with wing size [59]. There are also examples in *Drosophila* flies of no association between wing size and temperature variation (13 °C and 22 °C) [60].

In *M. spinolai* (sub *Triatoma spinolai* [61]) collected from various sites in northern Chile, adults from coastal populations were always wingless, while inland populations showed both wing and wingless adults. Winged males would be able to disperse, although this event does not guarantee that fliers will land in favourable sites for feeding and/or mating [61]. Brachyptery in *T. guasayana* is a heritable trait for at least three laboratory generations [35]. In other Hemiptera, the genetic base of brachytherapy has been found to be polygenic [62]. The morphological characteristics of the heads and wings observed in the Cochabamba population suggest a lack of dispersal by flight; therefore, the population would be genetically isolated. The differentiation observed in all of the characters would support this assumption, especially for those characters related to shape variation, which suggest genetic differentiation and, as mentioned above, this genetic differentiation between populations could be indicating different modes of dispersion. On the other hand, our results show that both head shape and wing shape correlate with geographic and climatic variables. Other insect species, such as *Drosophila subobscura*, or the fly *Scathophaga stercoraria*, also show a geographic pattern of morphological differentiation, including significant clinal variation in wing shape [52, 60].

## Conclusion

This study indicates that wing shape and head shape are the better markers for differentiated morphological variation across populations. Head measurements also varied across populations. Pronotum size is not necessarily a good indicator of muscle development and was found not to be directly related to body size in this species. Future studies should include genetic variation across populations and explore whether the brachypterous population indeed has limited genetic exchange.

## Supplementary Information

**Additional file 1:** **Table S1.** Eigenvalues and percentage of total variance explained by a principal component analysis of geographic and environmental factors from the T. guasayana populations studied. **Table S2.** Reclassification of *T. guasayana* individuals with the number and percentage of assigned individuals derived from discriminant function analyses performed for wings and heads. **Table S3.** Procrustes distances between pairs of *T. guasayana* populations performed for shape variables of wing and heads.

## Data Availability

The datasets supporting the conclusions of this article are included in the article and its additional files. Raw data are available from the corresponding author on reasonable request.

## References

[1] West-Eberhard MJ (2003). Developmental plasticity and evolution.

[2] Thompson DB, Foster S, Endler J (1999). Different spatial scales of natural selection and gene flow: the evolution of behavioral geographic variation and phenotypic plasticity. Geographic diversification of behavior: an evolutionary perspective.

[3] WHO. Chagas disease (also known as American trypanosomiasis). 2021. https://www.who.int/es/news-room/fact-sheets/detail/chagas-disease-(american-trypanosomiasis). Accessed 27 May 2022.

[4] de Paiva VF, Belintani T, de Oliveira J, Galvão C, da Rosa JA (2022). A review of the taxonomy and biology of *Triatominae* subspecies (*Hemiptera*: *Reduviidae*). Parasitol Res.

[5] Schofield CJ, Galvão C (2009). lassification, evolution and species groups within the *Triatominae*. Acta Trop.

[6] Cavallo MJ, Amelotti I, Gorla DE (2016). Invasion of rural houses by wild Triatominae in the arid Chaco. J Vector Ecol.

[7] Brito RN, Gorla DE, Diotaiuti L, Gomes ACF, Souza RCM, Abad-Franch F (2017). Drivers of house invasion by sylvatic Chagas disease vectors in the Amazon-Cerrado transition: a multi-year, state-wide assessment of municipality-aggregated surveillance data. PLoS Negl Trop Dis.

[8] Cardozo M, Fiad FG, Crocco LB, Gorla DE (2021). Triatominae of the semi-arid Chaco in Central Argentina. Acta Trop.

[9] Wisnivesky-Colli C, Schweigmann NJ, Alberti A, Pietrokovsky SM, Conti O, Montoya S (1992). Sylvatic American trypanosomiasis in Argentina. *Trypanosoma cruzi* infection in mammals from the Chaco forest in Santiago del Estero. Trans R Soc Trop Med Hyg.

[10] Rodríguez CS, Crocco LB, Nattero J (2004). Competencia vectorial de *Triatoma guasayana* (*Hemiptera*: *Reduviidae*): patrón de alimentación y excreción. Rev Soc Entomol Arg.

[11] Loza-Murguía M, Noireau F (2010). Vectorial capacity of *Triatoma guasayana* (Wygodzinsky & Abalos) (*Hemiptera*: *Reduviidae*) compared with two other species of epidemic importance. Neotrop Entomol.

[12] Abrahan LB, Gorla DE, Catalá SS (2011). Dispersal of *Triatoma infestans* and other *triatominae* species in the arid Chaco of Argentina—Flying, walking or passive carriage? The importance of walking females. Mem Inst Oswaldo Cruz.

[13] Lucero DE, Ribera W, Pizarro JC, Plaza C, Gordon LW, Peña R (2014). Sources of blood meals of sylvatic *Triatoma guasayana* near Zurima, Bolivia, assayed with qPCR and 12S cloning. PLoS Negl Trop Dis.

[14] Carcavallo RU, Canale DM, Martínez A (1988). Habitats de triatominos argentinos y zonas ecológicas donde prevalecen. Chagas.

[15] Canale DM, Cecere MC, Chuit R, Gurtler RE (2000). Peridomestic distribution of *Triatoma garciabesi* and *Triatoma guasayana* in northwest Argentina. Med Vet Entomol.

[16] Rodríguez-Planes LI, Vazquez-Prokopec GM, Cecere MC, Canale DM, Gürtler RE (2016). Selective insecticide applications directed against *Triatoma infestans* (*Hemiptera*: *Reduviidae*) affected a nontarget secondary vector of chagas disease Triatoma garciabesi. J Med Entomol.

[17] Dujardin JP, Schofield CJ, Panzera F. Les vecteurs de la maladie de Chagas: recherches taxonomiques, biologiques et génétiques. 2000. Brussels: Académie royale des sciences d'outre-mer. https://horizon.documentation.ird.fr/exl-doc/pleins_textes/2021-08/010023185.pdf.

[18] Almeida CE, Marcet PL, Gumiel M, Takiya DM, Cardozo-de-Almeida M, Pacheco RS (2009). Phylogenetic and phenotypic relationships among *Triatoma carcavalloi* (*Hemiptera*: *Reduviidae*: *Triatominae*) and related species collected in domiciles in Rio Grande do Sul State Brazil. J Vector Ecol.

[19] Justi SA, Russo CA, Mallet JRDS, Obara MT, Galvão C (2014). Molecular phylogeny of *Triatomini* (*Hemiptera*: *Reduviidae*: *Triatominae*). Parasit Vectors.

[20] Gorla D, Noireau F, Telleria J, Tibayrenc M (2017). Geographic distribution of Triatominae vectors in America. American trypanosomiasis Chagas disease.

[21] Wisnivesky-Colli C, Gürtler RE, Solarz ND, Schweigmann NJ, Pietrokovsky SM, Alberti A (1993). Dispersive flight and house invasion by *Triatoma guasayana* and *Triatoma sordida* in Argentina. Mem Inst Oswaldo Cruz.

[22] Noireau F, Dujardin JP (2001). Flight and nutritional status of sylvatic *Triatoma sordida* and *Triatoma guasayana*. Mem Inst Oswaldo Cruz.

[23] Lehane MJ, McEwen PK, Whitaker CJ, Schofield CJ (1992). The role of temperature and nutritional status in flight initiation by *Triatoma infestans*. Acta Trop.

[24] Schofield CJ, Lehane M, McEwen P, Catalá SS, Gorla DE (1992). Dispersive flight by *Triatoma infestans* under natural climatic conditions in Argentina. Med Vet Entomol.

[25] McEwen P, Lehane M (1993). Factors influencing flight initiation in the triatomine bug *Triatoma sordida* (*Hemiptera*: *Reduviidae*). Insect Sci Applic.

[26] McEwen P, Lehane M, Whitaker C (1993). The effect of adult population density on flight initiation in *Triatoma infestans* (Klug) (*Hemiptera*: *Reduviidae*). J Appl Entomol.

[27] Harrison RG (1980). Dispersal polymorphisms in insects. Annu Rev Ecol Syst.

[28] Gurevitz JM, Kitron U, Gürtler RE (2007). Flight muscle dimorphism and heterogeneity in flight initiation of field-collected *Triatoma infestans* (*Hemiptera*: *Reduviidae*). J Med Entomol.

[29] Hernández ML, Dujardin JP, Gorla DE, Catalá SS (2015). Can body traits, other than wings, reflect the flight ability of *Triatominae* bugs?. Rev Soc Bra Med Trop.

[30] Hernández ML, Espinoza J, Gomez M, Gorla D (2020). Morphological changes associated with brachypterous *Triatoma guasayana* (*Hemiptera*, *Reduviidae*) and their relationship with flight. Int J Trop Insect Sci.

[31] Almeida CE, Oliveira HL, Correia N, Dornak LL, Gumiel M, Neiva VL (2012). Dispersion capacity of *Triatoma sherlocki*, *Triatoma juazeirensis* and laboratory-bred hybrids. Acta Trop.

[32] Lawrence MG (2005). The relationship between relative humidity and the dewpoint temperature in moist air: a simple conversion and applications. Bull Am Meteorol Soc.

[33] Johnson CG (1969). Migration and dispersal of insects by flight.

[34] Ekkens DB (1981). Nocturnal flights of *Triatoma* (*Hemiptera*: *Reduviidae*) in Sabino Canyon, Arizona: I light collections. J Med Entomol.

[35] Dryden IL, Mardia KV (1998). Statistical shape analysis.

[36] Klingenberg CP (2011). MorphoJ: an integrated software package for geometric morphometrics. Mol Ecol Res.

[37] Di Rienzo JA, Casanoves F, Balzarini MG, Gonzalez L, Tablada M, Robledo CW (2016). InfoStat versión 2016 Universidad Nacional de Córdoba.

[38] Lachenbruch PA (1967). An almost unbiased method of obtaining confidence intervals for the probability of misclassification in discriminant analysis. Biometrics.

[39] Kumar S, Stecher G, Li M, Knyaz C, Tamura K (2018). MEGA X: molecular evolutionary genetics analysis across computing platforms. Mol Biol Evol.

[40] Ascarrunz E, Claude J, Joyce WG (2019). Estimating the phylogeny of geoemydid turtles (Cryptodira) from landmark data: an assessment of different methods. PeerJ.

[41] Rosenberg MS, Anderson CD (2011). PASSaGE: pattern analysis, spatial statistics, and geographic exegesis. version 2. Methods Ecol Evol..

[42] Le S, Josse J, Husson F (2008). FactoMineR: an R package for multivariate analysis. J Stat Softw.

[43] Bai Y, Dong JJ, Guan DL, Xie JY, Xu SQ (2016). Geographic variation in wing size and shape of the grasshopper *Trilophidia annulata* (*Orthoptera*: *Oedipodidae*): morphological trait variations follow an ecogeographical rule. Sci Rep.

[44] Noireau F, Flores R, Vargas F (1999). Trapping sylvatic *Triatominae* (*Reduviidae*) in hollow trees. Trans R Soc Trop Med Hyg.

[45] Hill JK, Thomas CD, Blakeley DS (1999). Evolution of flight morphology in a butterfly that has recently expanded its geographic range. Oecologia.

[46] Hughes CL, Dytham C, Hill JK (2007). Modelling and analysing evolution of dispersal in populations at expanding range boundaries. Ecol Entomol.

[47] Norberg U, Leimar O (2002). Spatial and temporal variation in flight morphology in the butterfly *Melitaea cinxia* (*Lepidoptera*: *Nymphalidae*). Biol J Lin Soc.

[48] Sekar S (2012). A meta-analysis of the traits affecting dispersal ability in butterflies: can wingspan be used as a proxy?. J Anim Ecol.

[49] Gutierrez D, Rosa M (1997). Patterns in the distribution, abundance and body size of carabid beetles (*Coleoptera*: *Carabidae*) in relation to dispersal ability. J Biogeogr.

[50] Dujardin JP, Costa J, Bustamante D, Jaramillo N, Catalá SS (2009). Deciphering morphology in *Triatominae*: the evolutionary signals. Acta Trop.

[51] O’Higgins P, Cobb SN, Fitton LC, Gröning F, Phillips R, Liu J (2011). Combining geometric morphometrics and functional simulation: an emerging toolkit for virtual functional analyses. J Anat.

[52] Taylor CP. Contribution of compound eyes and ocelli to steering of locusts in flight: I. behavioural analysis. J Exp Biol. 1981;93:1–18. 10.1242/jeb.93.1.1.

[53] Moser JC, Reeve JD, Bento JMS, Della Lucia TM, Cameron RS, Heck NM (2004). Eye size and behaviour of day-and night-flying leaf cutting ant alates. J Zool.

[54] Taylor GK, Krapp HG. Sensory systems and flight stability: what do insects measure and why? Insect Physiol. 2007;34:231–316. 10.1016/S0065-2806(07)34005-8.

[55] Suarez-Tovar CM, Sarmiento C (2016). Beyond the wing planform: morphological differentiation between migratory and nonmigratory dragonfly species. J Evol Biol.

[56] Klingenberg CP. Developmental constraints, modules and evolvability. In: Hallgrímsson B, Hall BK, editors. Variation: a central concept in biology. Amsterdam: Academic Press; 2005. p. 219–47.

[57] Ghalambor CK, McKay JK, Carroll SP, Reznick DN (2007). Adaptive versus non-adaptive phenotypic plasticity and the potential for contemporary adaptation in new environments. Funct Ecol.

[58] Schmid M, Guillaume F (2017). The role of phenotypic plasticity on population differentiation. Heredity.

[59] Schäfer MA, Berger D, Rohner PT, Kjaersgaard A, Bauerfeind SS, Guillaume F (2018). Geographic clines in wing morphology relate to colonization history in new world but not old world populations of yellow dung flies. Evolution.

[60] Santos M, Brites D, Laayouni H (2006). Thermal evolution of pre-adult life history traits, geometric size and shape, and developmental stability in *Drosophila subobscura*. J Evol Biol.

[61] Schofield CJ, Apt W, Sagua H, Panzera F, Dujardin JP (1998). Alary polymorphism in *Triatoma*
*spinolai* and its possible relationship with demographic strategy. Med Vet Entomol.

[62] Roff DA (1986). The evolution of wing dimorphism in insects. Evolution.

[63] Carcavallo RU, Curto de Casas SI, Sherlock IA, Galíndez Girón I, Jurberg J, Galvão C, et al. Geographical distribution and alti-latitudinal dispesion. In: Lent H, Carcavallo RU, Galíndez Girón I, Jurberg J, editors. Atlas of Chagas disease vectors in the Americas. Rio de Janeiro: Fiocruz Editorial; 1999. p. 747–92.

