# Supplementary material for: Phenotypic variability in traits related to flight dispersal in the wing dimorphic species Triatoma guasayana
Source: Parasit Vectors. 2023 Jan 9;16:8. doi: 10.1186/s13071-022-05570-7 (PMC9830765; doi:10.1186/s13071-022-05570-7)
Supplement: Supplementary file 1 — Additional file 1: Table S1. Eigenvalues and percentage of total variance explained by a principal component analysis of geographic and environmental factors from the T. guasayana populations studied. Table S2. Reclassification of T. guasayana individuals with the number and percentage of assigned individuals derived from discriminant function analyses performed for wings and heads. Table S3. Procrustes distances between pairs of T. guasayana populations performed for shape variables of wing and heads. [file 13071_2022_5570_MOESM1_ESM.docx]

**Additional file 1.**

**Table S1.**Eigenvalues, percentage of total variance explained, derived from a principal component analysis of geographic and climatic factors from the studied *T. guasayana* populations.

| Geographic and climatic variables | PCA axes | |
| --- | --- | --- |
|  | PC1 | PC2 |
| Eigenvalues | 3.583 | 2.141 |
| % total variance explained | 59.717 | 35.688 |
| Latitude | 0.792 | 0.561 |
| Longitude | -0.118 | 0.958 |
| Altitude | 0.811 | -0.571 |
| Temperature | -0.806 | 0.573 |
| Rainfall | 0.858 | 0.456 |
| Relative humidity (RH) | 0.947 | 0.214 |

**Table S2.** Reclassification of *Triatoma guasayana* populations performed for shape variation of wing, head and pronotum. The number of assigned individuals derived from discriminant function analyses is presented.

Population abbreviations: 12 J: 12 de Junio, C.A: Campo Alegre, Co: Cochabamba, C.E: Cruz del Eje, Hick: Hickman, Indep: Independencia, Isch: Ischilín, Lor: Loreto, S.M: San Martín, V.F: Valle Fértil.

|  | Module | Nº of individuals | 12 J. | C.A | Co | C.E | Hick | Indep | Isch | Lor | S.M | V.F | Error (%) |
| --- | --- | --- | --- | --- | --- | --- | --- | --- | --- | --- | --- | --- | --- |
| 12 J | Wing | 7 | 6 | 0 | 0 | 0 | 0 | 0 | 0 | 0 | 1 | 0 | 14.29 |
|  | Head | 7 | 1 | 2 | 0 | 0 | 0 | 0 | 1 | 3 | 0 | 0 | 85.71 |
| C.A | Wing | 4 | 0 | 4 | 0 | 0 | 0 | 0 | 0 | 0 | 0 | 0 | 0 |
|  | Head | 5 | 0 | 3 | 0 | 1 | 0 | 0 | 0 | 1 | 0 | 0 | 40 |
| Co | Wing | 5 | 0 | 0 | 5 | 0 | 0 | 0 | 0 | 0 | 0 | 0 | 0 |
|  | Head | 16 | 0 | 0 | 16 | 0 | 0 | 0 | 0 | 0 | 0 | 0 | 0 |
| C.E | Wing | 5 | 0 | 0 | 0 | 5 | 0 | 0 | 0 | 0 | 0 | 0 | 0 |
|  | Head | 5 | 0 | 0 | 0 | 3 | 0 | 0 | 1 | 1 | 0 | 0 | 20 |
| Hick | Wing | 5 | 0 | 0 | 0 | 0 | 4 | 0 | 1 | 0 | 0 | 0 | 20 |
|  | Head | 5 | 0 | 0 | 0 | 0 | 4 | 0 | 0 | 1 | 0 | 0 | 20 |
| Indep | Wing | 8 | 0 | 0 | 0 | 0 | 0 | 6 | 0 | 0 | 1 | 1 | 25 |
|  | Head | 7 | 0 | 0 | 0 | 0 | 0 | 5 | 0 | 0 | 2 | 0 | 7 |
| Isch | Wing | 4 | 0 | 0 | 0 | 0 | 0 | 0 | 4 | 0 | 0 | 0 | 0 |
|  | Head | 4 | 0 | 0 | 0 | 0 | 0 | 0 | 4 | 0 | 0 | 0 | 0 |
| Lor | Wing | 6 | 0 | 0 | 0 | 0 | 0 | 0 | 0 | 5 | 1 | 0 | 16.67 |
|  | Head | 6 | 1 | 0 | 0 | 0 | 2 | 0 | 0 | 3 | 0 | 0 | 50 |
| S.M | Wing | 5 | 1 | 0 | 0 | 1 | 0 | 0 | 0 | 1 | 2 | 0 | 60 |
|  | Head | 6 | 0 | 0 | 0 | 0 | 0 | 1 | 0 | 0 | 3 | 2 | 50 |
| V.F | Wing | 3 | 0 | 0 | 0 | 0 | 0 | 0 | 0 | 0 | 0 | 3 | 0 |
|  | Head | 3 | 0 | 0 | 0 | 0 | 0 | 0 | 0 | 0 | 1 | 2 | 33.33 |

**Table S3** Procrustes distances between pairs of population of *T. guasayana* performed for shape variables of wing and head. Upper and lower diagonals correspond to wings and heads, respectively.

Population abbreviations: 12 J: 12 de Junio, C.A: Campo Alegre, Co: Cochabamba, C.E: Cruz del Eje, Hick: Hickman, Indep: Independencia, Isch: Ischilín, Lor: Loreto, S.M: San Martín, V.F: Valle Fértil.

|  | 12 J. | C.A | Co | C.E | Hick | Indep | Isch | Lor | S.M | V.F |
| --- | --- | --- | --- | --- | --- | --- | --- | --- | --- | --- |
| 12 J. | - | 0.041^**^ | 0.042^**^ | 0.051^***^ | 0.028 | 0.030^*^ | 0.042^**^ | 0.039^***^ | 0.027 | 0.029 |
| C.A | 0.012 | - | 0.049^**^ | 0.053^**^ | 0.030 | 0.042^***^ | 0.020 | 0.024 | 0.030 | 0.037 |
| Co | 0.096^***^ | 0.096^***^ | - | 0.059^**^ | 0.049^**^ | 0.049^**^ | 0.05^**^ | 0.043^**^ | 0.05^**^ | 0.044^*^ |
| C.E | 0.018 | 0.025 | 0.101^***^ | - | 0.051^**^ | 0.029^***^ | 0.042^*^ | 0.037^**^ | 0.039^*^ | 0.040^*^ |
| Hick | 0.03^*^ | 0.033 | 0.091^***^ | 0.031^**^ | - | 0.036^**^ | 0.031 | 0.027 | 0.029 | 0.026 |
| Indep | 0.103^***^ | 0.106^**^ | 0.044^***^ | 0.108^***^ | 0.088^***^ | - | 0.030^**^ | 0.034^**^ | 0.017 | 0.021 |
| Isch | 0.018 | 0.019 | 0.107^***^ | 0.016 | 0.036^**^ | 0.115^**^ | - | 0.023 | 0.023 | 0.028 |
| Lor | 0.024 | 0.031^*^ | 0.091^***^ | 0.025^*^ | 0.013 | 0.09^***^ | 0.032^*^ | - | 0.031 | 0.030 |
| S.M | 0.01^***^ | 0.103^**^ | 0.045^***^ | 0.104^***^ | 0.084^**^ | 0.009 | 0.112^*^ | 0.086^**^ | - | 0.020 |
| V.F | 0.105^**^ | 0.107^*^ | 0.042^***^ | 0.109^*^ | 0.089^*^ | 0.013 | 0.116^*^ | 0.091^**^ | 0.010 | - |

***** *P* < 0.05 ** *P* < 0.01 *** *P* < 0.001
